# Sexual prejudice declined across generational cohorts and genders: A cohort sequential latent growth curve model from 2014 to 2024

**DOI:** 10.1111/bjso.70087

**Published:** 2026-04-28

**Authors:** Eden V. Clarke, Chris G. Sibley, Danny Osborne

**Affiliations:** ^1^ School of Psychology University of Auckland Auckland New Zealand

**Keywords:** gender differences, LGBTQIA+, lifespan development, longitudinal modelling, sexual prejudice

## Abstract

Despite attitudes towards the LGBTQIA+ community improving in recent years, older (vs younger) cohorts still report higher rates of sexual prejudice. To date, it is unclear if this generational difference emerges due to normative ageing or the distinct social norms in which each generation was born and raised (cohort effects). This pre‐registered study clarifies the issue by utilizing cohort sequential latent growth curve modelling to examine the developmental trajectory of sexual prejudice for men and women across 11 annual waves of longitudinal panel data (*N* = 63,558). Our results reveal a period effect in which older (vs younger) cohorts and men (vs women) display higher initial mean levels of sexual prejudice. But due to shared social conditions, most cohorts experience comparable curvilinear declines in sexual prejudice across time. Collectively, our results highlight the malleability of sexual prejudice across the lifespan and demonstrate the need to examine the socio‐political environment when taking a lifespan development perspective on anti‐LGBTQIA+ attitudes.

## INTRODUCTION

Support for the LGB+ community has increased considerably during the 21st century. For example, whereas same‐sex marriage was a ‘hot‐button’ issue in the early 2000s, support for same‐sex relationships is now a hallmark of Western democracies (Baunach, 2012; Brickell, 2020; Kite, 2011). Indeed, same‐sex relationships are decriminalized in over 100 countries (ILGA, 2025), with same‐sex marriage and adoption being legal in over 30 countries (Equaldex, 2025). Beyond legal protections, social acceptance of the LGBTQIA+ community has grown in recent years with a remarkable rise in pro‐LGBTQIA+ campaigns, art, music and media (Kite, 2011; McInroy & Shelley, 2017; Nölke, 2018). It is perhaps unsurprising, then, that scholars have grown optimistic about the progress of LGBTQIA+ rights, with emerging research demonstrating that support for same‐sex relationships is steadily increasing over time (Baunach, 2012; Kreitzer et al., 2014).

Despite this promising trend towards equality, the existing scholarship reveals a generational divide whereby older cohorts have higher levels of sexual prejudice than their younger counterparts (Herek, 2009; Herek & McLemore, 2013). To date, it remains unclear exactly *why* this trend occurs. One perspective suggests that this generational gap is due to normative ageing and that people simply become more conservative as they age (e.g., Peterson et al., 2020; Wilson, 1996). However, an alternative scholarship posits that differences between generations occur due to distinct socialization experiences (e.g., Crandall et al., 2018). Because ageing and social changes unfold simultaneously, fully distinguishing between these two processes requires large‐scale longitudinal data that captures the lifespan development of sexual prejudice over time.

To these ends, the current pre‐registered study utilizes 11 annual waves of longitudinal panel data spanning 10 years to assess whether changes in sexual prejudice reflect normative developmental processes (ageing effect), a general trend across time among birth cohorts (period effect) or enduring differences between birth cohorts (e.g., cohort effect). We begin by unpacking the ageing perspective. Next, we highlight the impact of egalitarian social shifts on prejudiced attitudes and the impressionable years hypothesis. We conclude by highlighting the importance of our unique analytic approach and the hypotheses of the current study.

## THE DEVELOPMENT OF SEXUAL PREJUDICE OVER TIME

### Ageing effects

Scholars initially assumed that generational differences in sexual prejudice emerge because people become more prejudiced as they age (Peterson et al., 2020; Wilson, 1996). Indeed, the ageing process encompasses various psychological, social, economic and biological changes that are thought to affect people's prejudiced attitudes (Peterson et al., 2020). For example, normative ageing spans work, familial and financial stability, as well as declines in physical health and cognition (Sears, 1981; St. Jacques et al., 2009). Thus, as people age, tolerance for uncertainty, openness and contact with outgroups often decline (Jost et al., 2007; Peterson et al., 2020). Moreover, death anxiety and preferences for order, security and hierarchy increase (Jost et al., 2007). Because uncertainty avoidance and threat management predict conservative values (Jost et al., 2007), changes in sexual prejudice over time may reflect normative ageing processes. According to this perspective, people at similar life stages will share the same level of sexual prejudice, irrespective of when they were born. For instance, a 50‐year‐old in 2014 would report the same levels and rate of change in sexual prejudice as a 50‐year‐old in 2024.

### Egalitarian socialization

Although normative ageing has been a dominant explanation for generational differences in prejudice over the past decades, an alternative perspective suggests that the differences between younger and older generations reflect distinct socialization experiences (Zubielevitch et al., 2023). That is, social shifts which emphasize the importance of LGBTQIA+ rights are anticipated to impact people's sexual prejudice. In particular, the 1960s‐80s reflect a period of heightened proliferation of LGBTQIA+ rights, especially in New Zealand (i.e., the location of the present study). Of note is the formation of gay social clubs (1960s), LGBTQIA+ liberation groups (1970s), pro‐LGB+ protests (1970s‐80s), the first pride event (1972) and the decriminalization of homosexuality (1986). After this initial period of progress, Western democracies have continued to pass progressive legislation on civil union, same‐sex marriage, adoption, blood donations and conversion therapy (Brickell, 2020; Hansen, 2023; Neilson, 2022; Saxton, 2020). Because the political and social environment following the expansion of LGBTQIA+ rights is markedly different from prior decades (Hansen, 2023), cohorts who came of age during this period should notably differ from those raised in a different zeitgeist in their endorsement of sexual prejudice. Whether these cohort differences emerge in their initial mean levels (e.g., period effect) or their rates of change (e.g., cohort effect) in sexual prejudice, however, remains unknown.

### Period effects

One way in which socialization could contribute to generational differences in sexual prejudice is through period effects. Indeed, numerous scholars argue that prejudiced attitudes are continuously updated in response to changes in group norms (Álvarez‐Benjumea, 2025; Arnold et al., 2026; Crandall et al., 2018; Sherif & Sherif, 1953). For example, the lifelong openness model argues that attitude stability in older cohorts is not due to an inability to change, but rather, lifestyle constraints provide fewer *opportunities* for change (Miller & Sears, 1986; Sears, 1983; Tyler & Schuller, 1991). Accordingly, when exposed to discrepant experiences or social norms, older cohorts change their attitudes to reflect the new norm, often at the same rate as their younger counterparts (Miller & Sears, 1986; Tyler & Schuller, 1991). Consistent with this perspective, the legalization of same‐sex relationships predicted an increase in acceptance of the LGBTQIA+ community in Canada (Matthews, 2005) and numerous countries across Europe (Aksoy et al., 2020). A growing scholarship (e.g., Brown & Paterson, 2016) also reveals that positive exposure to LGBTQIA+ people in the media predicts declines in sexual prejudice via vicarious contact in both the US (Preuß & Steffens, 2021) and Italy (Vezzali et al., 2015). Taken together, these studies suggest that, although cohorts may have different initial mean levels of sexual prejudice, they should exhibit the same trajectory of change in sexual prejudice over time (i.e., a period effect) due to shared social conditions and conformity to new group norms. Fully explicating this possibility, however, requires longitudinal data that adjusts for normative ageing and cohort effects.

### Cohort effects

Whereas a period effect assumes prejudiced attitudes are malleable across time, a divergent scholarship argues that prejudices are developed and crystallized in people's formative years. Indeed, the impressionable years' hypothesis posits that prejudiced attitudes are most pliable during childhood and early adulthood but become rigid thereafter (Alwin et al., 1991; Osborne et al., 2011; Sears, 1981). As such, the environment and group norms that people were exposed to in their formative years are expected to have a lasting impact on their prejudiced attitudes. In the case of sexual prejudice, older generations were raised when homosexuality was criminalized, and sexual prejudice was normalized (Hansen, 2023). In contrast, younger cohorts were socialized with the proliferation of legal and social protections for LGBTQIA+ people (Clarke et al., 2025; Hansen, 2023). If attitudes do crystallize in early adulthood, then older cohorts should be more prejudiced and stable in their attitudes over time, compared with younger cohorts. Accordingly, Kreitzer et al. (2014) found that younger people are more likely than older people to increase their support for same‐sex marriage following its legalization in the US. Likewise, Clarke, Lilly, et al. (2026) examined the trajectory of support for same‐sex relationships across 14 years and revealed that, although the New Zealand population is increasing in support for same‐sex relationships, older participants were slower to change than their younger counterparts. Consistent with the impressionable years' hypothesis, these findings indicate that older cohorts may have already cemented their attitudes towards the LGBTQIA+ community and are thus more resistant than their younger counterparts to change over time.

### Gender differences in sexual prejudice over time

In addition to generational differences, the growth trajectory of sexual prejudice may differ among men and women. Indeed, it is well documented that men are higher in sexual prejudice than women (Herek, 1988; Kite & Whitley, 1996, 1997; LaMar & Kite, 1998). Kite and Whitley (1997) argue that heterosexual men are higher in sexual prejudice because LGBTQIA+ identities threaten traditional forms of masculinity and the associated social hierarchy that places men above women and sexual minorities. In other words, heterosexual men (but not women) are expected to condemn LGBTQIA+ identities because doing so emphasizes their masculinity and power within the patriarchy.

A related literature demonstrates that the socialization of masculine norms during men's formative years is particularly rigid and, therefore, may undermine changes in sexual prejudice over time (Pascoe, 2005; Poteat & Anderson, 2012). In contrast, women are socialized with feminine gender roles which are (relatively) more flexible and, thus, open to change over time (LaMar & Kite, 1998; Poteat & Anderson, 2012). Consistent with this thesis, Poteat and Anderson (2012) reveal that adolescent boys showed no significant changes in their attitudes towards gay men over time despite declines in adolescent girls' sexual prejudice. Thus, men will likely display higher levels and slower rates of change in sexual prejudice than women. Prior work on the developmental trends in sexual prejudice among men and women has, however, exclusively focused on adolescence (e.g., Poteat & Anderson, 2012). As such, it remains unknown how sexual prejudice develops across the full adult lifespan (e.g., 18–84 years).

## THE CURRENT STUDY

In sum, there are three competing explanations for the generational differences in sexual prejudice: ageing, period and cohort differences. Elucidating these developmental trajectories is, however, difficult given that ageing, cohort and period effects unfold simultaneously. Indeed, properly separating the unique contribution of these processes requires age‐specific longitudinal data where participants comprise distinct generations and each generational cohort overlaps in age (O'Donnell et al., in press; Prinzie & Onghena, 2005; Zubielevitch et al., 2023). Notably, these data must differentiate between cohorts who came of age with the expansion of LGBTQIA+ rights and those who did not. Perhaps due to the difficulty in obtaining large‐scale longitudinal panel data that spans meaningful social change, no research to date has explicated how sexual prejudice develops across the adult lifespan for distinct cohorts.

The current pre‐registered study addresses this oversight by leveraging 11 annual waves of longitudinal panel data (2014–2024) from New Zealand to perform a cohort‐sequential latent growth curve model. This analytical approach allows us to separate ageing and societal trajectories by examining the rate of change in sexual prejudice among 12 different birth cohorts spanning 1995–1936 (ages 19–84). More specifically, we estimate three separate cohort‐sequential latent growth curve models that make different assumptions about the developmental trends of sexual prejudice over time. These models assume that sexual prejudice reflects (a) normative development across the lifespan (ageing effect), (b) a general trend across time among birth cohorts (period effect) or (c) differences between birth cohorts due to the distinct social context in which each birth cohort reached maturity (cohort effects). Due to the documented differences in the mean levels and rates of change in sexual prejudice between men and women (Kite & Whitley, 1997; Poteat & Anderson, 2012), we also examine whether there are gender differences in the trajectory of sexual prejudice across time. Importantly, this approach will allow us to clarify whether the trajectories of sexual prejudice are different among groups who experienced substantially different forms of gender role socialization.

### Hypotheses

Although there are competing explanations for how sexual prejudice develops over the adult lifespan, the impressionable years hypothesis has (arguably) received the most support over the recent decades (e.g., Poteat & Anderson, 2012). Thus, given that prejudiced attitudes are shaped by social norms (Matthews, 2005), particularly those present during people's impressionable years (Alwin et al., 1991; Osborne et al., 2011; Sears, 1981), we expect to find cohort‐based differences in sexual prejudice over time for women and men (Hypothesis 1). To elaborate, since the early 1960s, there has been a remarkable rise in LGB+ rights movements and legislation (e.g., the same‐sex *Marriage Amendment Act* in 2013). These events should have a unique impact on cohorts who witnessed this proliferation of LGB+ rights as they came of age. Additionally, because men (vs women) are typically higher on sexual prejudice (Herek, 1988; Kite & Whitley, 1997) and more resistant to change (Poteat & Anderson, 2012), we expect that men of all cohorts will exhibit higher mean levels and slower rates of change in sexual prejudice over time than women (Hypothesis 2).

## METHODS

### Data and materials

The design and analysis for the current study were pre‐registered^1^ on the Open Science Framework (OSF): https://osf.io/x4mb5. Data for the current study came from the New Zealand Attitudes and Values Study (NZAVS). The NZAVS was approved by the University of Auckland Human Participants Ethics Committee (reference number: UAHPEC22576), and informed consent was given by participants. Participants' responses are anonymised and confidential. Our ethics approval specifies that we are unable to post our dataset online—doing so would violate the conditions of our ethics approval. However, a de‐identified dataset containing the variables analysed in this manuscript is available upon request from the corresponding author or any NZAVS advisory board member for replication purposes. The Mplus syntax used for all models reported in this study is also available on the OSF: https://osf.io/dz92p.

### Sampling procedure

The NZAVS is an ongoing longitudinal panel study of New Zealand adults that began in 2009. Participants were initially sampled from the New Zealand electoral roll (Time 1; *N* = 6518; response rate = 16.6%). Importantly, because voter registration is compulsory in New Zealand, this sample effectively constitutes a random sample of New Zealand adults. To address sample attrition and diversify the sample, nine additional booster samples were recruited in 2011 (Time 3; *N* = 6884, *n*booster = 2966), 2012 (Time 4; *N* = 12,179, *n*booster = 5107), 2013 (Time 5; *N* = 18,261, *n*booster = 7487), 2016 (Time 8; *N* = 21,936, *n*booster = 7667), 2018 (Time 10; *N* = 47,948, *n*booster = 29,921), 2019 (Time 11; *N* = 42,681, *n*booster = 4734), 2021 (Time 13; *N* = 34,131, *n*booster = 1301), 2022 (Time 14; *N* = 33,722, *n*booster = 1574) and 2023 (Time 15; *N* = 32,857, *n*booster = 3293). At Time 16, the sample size was 31,873 (70.27% retention from Time 15; 28.34% retention from Time 1). Sibley (2024) provides a full overview of the sampling procedure, booster sampling, ethics and retention rates (see the OSF: https://osf.io/75snb/).

### Participant details

The current study focuses on the 63,558 participants who provided partial or complete responses to our focal variables at one or more assessment occasions (see Table 1). Of these participants, 37.0% identified as men (*n* = 23,514), 63.0%% identified as women (*n* = 40,044). Concerning ethnicity, 78.2% identified as New Zealand European (*n* = 49,733), 12.4% as Māori (*n* = 7902), 2.6% as Pacific Nations ancestry (*n* = 1629), 5.3% as Asian ancestry (*n* = 3348) and 1.5% did not report their ethnicity (*n* = 946). The mean age of the sample was 46.68 (*SD* = 13.23) at Time 6 (i.e., the first assessment of sexual prejudice and, thus, the start of our study).

**TABLE 1:** Age and sample size for birth cohorts.

Birth cohorts	Age at time 6 (~2014)	Age at time 16 (~2024)	Women	Men
			*n*	*n*
1995–1991	19	29	2758	1343
1990–1986	24	34	3008	1449
1985–1981	29	39	3169	1522
1980–1976	34	44	3628	1808
1975–1971	39	49	4402	2232
1970–1966	44	54	4944	2756
1965–1961	49	59	5709	3438
1960–1956	54	64	5942	3993
1955–1951	59	69	4212	3177
1950–1946	64	74	1322	985
1945–1941	69	79	672	554
1940–1936	74	84	278	257
*n* group			40,044	23,514
*N*_total_			63,558	

*Note*: The youngest age in the birth cohort was used as an indication of participants' age at Time 6.

### Materials

All measures were embedded within a larger omnibus survey containing measures outside the scope of the present study.

**Sexual Prejudice** was assessed with a single item adapted from Pew Research Center (2008): “I think that homosexuality should be accepted by society” (reverse‐coded). This item is measured on a 1 (strongly disagree) to 7 (strongly agree) scale and was added to the NZAVS at Time 6 (i.e., in 2014).

**Gender** was assessed with an open‐ended item: “What is your gender?”. Gender was then coded into a dichotomous variable according to a scheme developed by the NZAVS (0 = woman, 1 = man; see Fraser et al., 2020). Although this open‐ended measure was developed as a gender‐inclusive way for participants to self‐identify with their preferred label(s), less than 1.0% of participants self‐identified as transgender, non‐binary or gender diverse (TGD). This sample size of TGD participants is, therefore, too small to be included in our analytic approach. As such, we focus on participants who consistently self‐identify as a “man” or “woman”. Nevertheless, because many members of the community do not self‐identify as transgender (i.e., self‐report as “man” or “woman”; see Fraser et al., 2020; Lilly et al., 2023), we are unable to distinguish between cisgender and binary transgender participants.

### Analytic approach

To examine whether changes in sexual prejudice are due to normative ageing, societal shifts or generational differences and whether these changes differed by men and women, we conducted a multi‐group cohort sequential latent growth curve model based on 5‐year birth cohorts in *Mplus* version 8.10 with full information maximum likelihood (FIML) estimation to account for missing data (also see Lilly et al., 2025; Zubielevitch et al., 2023). Like a latent growth model, a cohort sequential latent growth curve model estimates developmental trajectories over time. The cohort sequential latent growth curve model, however, extends the traditional approach by simultaneously estimating the intercepts and slopes of sexual prejudice for different cohorts. More specifically, we can estimate three cohort‐sequential models with different constraints to examine if changes in sexual prejudice reflect normative ageing (ageing effect), a general trend across time among birth cohorts (period effect) or differences between birth cohorts (cohort effect). By overlapping the estimates for each birth cohort, we can reveal common developmental trends and potential differences between cohorts across time.

We sort our sample into 5‐year birth cohorts based on their year of birth, with a total of 12 different birth cohorts spanning 1995–1936 (ages 19–84). We use the youngest possible age within each cohort as an indicator of age at Time 6 (i.e., our first assessment occasion, 2014). As such, the growth trajectories for each cohort reflect normative ageing across 10 years and changes from 2014 to 2024. In other words, the 1995–1990 birth cohort reflected 10 years of changes from ages 19–29, the 1990–1986 birth cohort reflected changes from ages 24–34 and so on. We also separated each birth cohort by gender to model separate trajectories in sexual prejudice for women and men.

We began by estimating an ageing model that assumes all generations, irrespective of when they were born, experience the same initial mean level and rate of change in sexual prejudice when they are the same age. That is, a 50‐year‐old at Time 6 (2014) would report the same levels and rate of change in sexual prejudice as a 50‐year‐old at Time 16 (2024). As such, we constrained all intercepts and slopes to equality across all birth cohorts. Importantly, to account for curvilinear change over time, we estimated both linear and quadratic components in our analyses. This model was centred at the mid‐point of our age range of interest (i.e., 45‐years) and conditioned by age to allow us to plot the point estimates of sexual prejudice on a continuum from ages 19–84. In doing so, we graphed the common growth trajectory in sexual prejudice across the adult lifespan by allowing each birth cohort to contribute to a different segment of the curve.

To account for the possibility that differences in sexual prejudice are due to societal trends, we then estimated a period model. The period model assumes that all birth cohorts differ in their initial mean level of sexual prejudice. But, due to shared social conditions (e.g., the legalization of same‐sex marriage), each birth cohort will exhibit the same rate of change in sexual prejudice over time. As such, we constrained the slopes but allowed the intercepts to vary across cohorts.

Finally, we estimated a cohort model that assumes that each birth cohort will differ in their initial mean and rate of change in sexual prejudice over time due to the different societal conditions under which each birth cohort reached maturity. In other words, those who came of age in the 1940s will exhibit substantially different mean levels of, and rates of change in, sexual prejudice than those who came of age in the 1990s because of the unique experiences (e.g., LGB+ rights movements or lack thereof) that occurred during each birth cohort's formative developmental years. As such, the intercepts and slopes were free to vary across cohorts. Like the ageing model, we conditioned the estimates by age in years to plot the trends for each birth cohort's age across the 11 annual assessment occasions. Accordingly, the growth trajectories for sexual prejudice for one birth cohort at the first half of their assessment points overlap with the previous birth cohort at the last half of their assessment points. Therefore, we can examine if the 95% error bars from these estimates overlap across the birth cohort; overlapping error bars would indicate that differences in sexual prejudice are attributable to normative ageing. In contrast, nonoverlapping error bars suggest distinct cohort differences in sexual prejudice.

To determine if changes in sexual prejudice reflect an ageing, period or cohort effect, we first compare the global fit statistics of the three models. These fit statistics include the comparative fit statistics (CFI; Wang & Wang, 2012), the standardized root mean squared residual (SRMR; Hu & Bentler, 1999), and the root mean square error of approximation (RMSEA; McDonald & Ho, 2002). We also report the Akaike Information Criterion (AIC), the Bayesian Information Criterion (BIC) and the sample‐size adjusted BIC (aBIC), whereby lower values indicate a better model fit (Muthén & Muthén, 2000). Finally, we report the chi‐squared test statistic, but its sensitivity to large samples renders it an impractical test of model fit (Wang & Wang, 2012). Because these fit statistics cannot fully elucidate important distinctions between ageing and cohort effects (Steiger, 2007), we also plot the estimates for the ageing and birth cohorts' models by age to visually inspect whether these estimates reflect normative ageing, cohort differences, or period effects.

## RESULTS

### Ageing model

**FIGURE 1:**
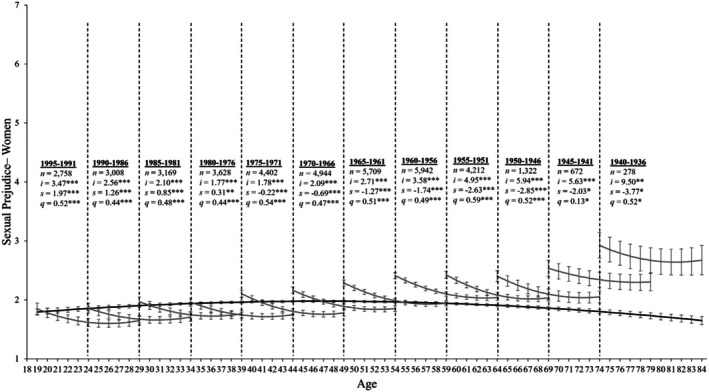
Change trajectories and comparison of ageing and cohort models for women's sexual prejudice. Change trajectories for women's sexual prejudice are shown in the back line from ages 18–84. The grey lines within each 5‐year birth cohort panel demonstrate longitudinal change in sexual prejudice over 11 assessments by estimating the latent intercept (*i*), linear slopes (*s*) and quadratic slopes (*q*) and overlap with subsequent birth cohorts by 6 years. The estimations are based on mean levels of sexual prejudice shown on the y‐axis across age (in years) and assessments (annual) on the x‐axis, with 95% confidence intervals as error bars around each point estimate. All birth cohorts show significant rates of change. *p ≤ .05, **p ≤ .01, ***p ≤ .001.

**FIGURE 2:**
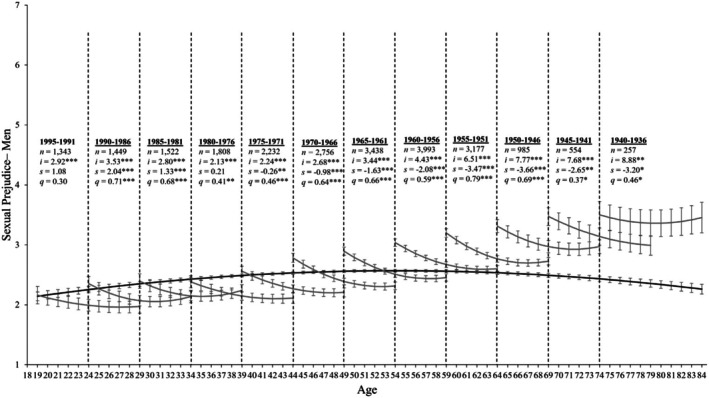
Change trajectories and comparison of ageing and cohort models for men's sexual prejudice. Change trajectories for men's sexual prejudice are shown in the back line from ages 18–84. The grey lines demonstrate longitudinal change in sexual prejudice over 11 assessments by estimating the latent intercept (*i*), linear slopes (*s*) and quadratic slopes (*q*) and overlap with subsequent birth cohorts by 6 years. The estimations are based on mean levels of sexual prejudice shown on the y‐axis across age (in years) and annual assessments on the x‐axis, with 95% confidence intervals as error bars around each point estimate. Birth cohorts with significant rates of change are underlined for clarity. *p ≤ .05, **p ≤ .01, ***p ≤ .001.

Except for the SRMR (SRMR = 0.107), the ageing model fit these data well, *χ* ^2^ (1839) = 24163.409, *p* < .001, CFI = .917, RMSEA = 0.068. Given that the SRMR deviates only slightly from conventional standards and that all other fit indices meet conventional cutoffs, we retain the ageing model. Consistent with this analytical choice, many scholars argue that the conventional cutoff standards proposed by Hu and Bentler (1999) are too stringent for complex models and should thus be used as guidelines rather than strict rules (Nye, 2023). With this in mind, the parameter estimates that best fit all birth cohorts are displayed in Table 2. For women, the ageing model suggests a curvilinear decrease in sexual prejudice (*s* = 0.009, *SE* = 0.005, *p* = .116; *q* = −0.024, *SE* = 0.003, *p* < .001; see Table 3). Indeed, the black line displayed in Figure 1 reveals that sexual prejudice slowly increased from age 19 until approximately age 40, where it stabilized until approximately age 60 before slowly declining until age 84. Turning to our results for men, sexual prejudice had a significant linear increase (*s* = 0.063, *SE* = 0.08, *p* < .001; see Table 3) that slowed over time (*q* = −0.034, *SE* = 0.003, *p* < .001). Similar to women, Figure 2 shows that men's sexual prejudice slowly increased from age 19 until approximately age 50 before declining thereafter.

**TABLE 2:** Model fit for ageing, period and cohort models.

Model	*χ* ^2^	*df*	*p*‐value	CFI	RMSEA	SRMR	AIC	BIC	aBIC
Ageing	24,163.409	1838	<.001	0.917	0.068	0.107	863,554.094	863,644.691	863,612.911
Period	20,780.234	1816	<.001	0.930	0.063	0.106	860,214.919	860,504.830	860,403.133
Cohort	19,805.555	1772	<.001	0.933	0.062	0.106	859,328.241	860,016.779	859,775.249

Model comparisons	Δ*χ* ^2^	Δ*df*	*p*‐value	ΔCFI	ΔRMSEA	ΔSRMR	ΔAIC	ΔBIC	ΔaBIC
Age–period	3383.175	22	<.001	−0.013	0.005	0.001	3339.175	3139.861	3209.778
Period–cohort	974.679	44	<.001	−0.003	0.001	0.000	886.678	488.051	627.884

Abbreviations: aBIC, sample‐size adjusted BIC; AIC, Akaike Information Criterion; BIC, Bayesian Information Criterion; CFI, Comparative Fit Index; *df*, degrees of freedom; RMSEA, root mean square error of approximation; SRMR, standardized root mean square residual; *χ* ^2^, chi‐squared.

**TABLE 3:** Parameter coefficients for the ageing model.

	Est.	*SE*	Est./*SE*	*p*‐value	95% CI		Variances
					LB	UB	
*Women*							
Intercept (*i*)	1.98	0.01	228.63	<.001	1.958	1.992	1.97
Linear slope (*s*)	0.01	0.01	1.57	.116	−0.002	0.019	0.13
Quadratic slope (*q*)	−0.02	0.00	−9.12	<.001	−0.029	−0.019	0.00
*Men*							
Intercept (*i*)	2.54	0.01	221.55	<.001	2.517	2.562	1.97
Linear slope (*s*)	0.06	0.01	8.40	<.001	0.049	0.078	0.13
Quadratic slope (*q*)	−0.03	0.00	−10.22	<.001	−0.041	−0.028	0.00

Abbreviations: CI, confidence interval; LB, lower bound; *SE*, standard error; UB, upper bound.

### Period model

The period model of sexual prejudice also fits these data well across all indices, but the SRMR once again fell slightly short of conventional standards, *χ* ^2^ (1816) = 20,780.234, *p* < .001, CFI = .930, RMSEA = 0.063, SRMR = 0.106. Table 4 reveals that the freely estimated intercepts were higher for each successive cohort of women and men. As such, older cohorts (e.g., those born in 1936) of men and women display a higher mean level of sexual prejudice than their younger counterparts (e.g., those born in 1995). The period model suggests a curvilinear decline in sexual prejudice over time for women (*s* = −0.243, *SE* = 0.009, *p* < .001; *q* = −0.014, *SE* = 0.003, *p* < .001) and men (*s* = −0.305, *SE* = 0.013, *p* < .001; *q* = −0.012, *SE* = 0.004, *p* = .005).

**TABLE 4:** Parameter estimates for the period model.

Birth cohort		Women						Men					
		Est.	*SE*	Est./*SE*	*p*‐value	95% CI		Est.	*SE*	Est./*SE*	*p*‐value	95% CI	
						LB	UB					LB	UB
1995–1991	Intercepts freely estimated	1.21	0.04	29.57	<.001	1.132	1.292	1.44	0.06	24.08	<.001	1.321	1.555
1990–1986		1.36	0.03	39.41	<.001	1.288	1.423	1.67	0.05	33.19	<.001	1.568	1.765
1985–1981		1.53	0.03	51.66	<.001	1.473	1.590	1.88	0.04	43.43	<.001	1.796	1.966
1980–1976		1.64	0.03	64.31	<.001	1.586	1.686	2.00	0.04	54.96	<.001	1.928	2.070
1975–1971		1.82	0.02	82.25	<.001	1.773	1.860	2.27	0.03	72.92	<.001	2.209	2.331
1970–1966		2.02	0.02	96.62	<.001	1.979	2.061	2.56	0.03	90.92	<.001	2.501	2.611
1965–1961		2.25	0.02	109.37	<.001	2.209	2.290	2.84	0.03	106.16	<.001	2.786	2.891
1960–1956		2.50	0.02	112.79	<.001	2.458	2.545	3.16	0.03	114.28	<.001	3.107	3.216
1955–1951		2.63	0.03	93.60	<.001	2.577	2.687	3.44	0.03	102.65	<.001	3.373	3.505
1950–1946		2.81	0.05	58.72	<.001	2.715	2.902	3.84	0.06	68.55	<.001	3.726	3.946
1945–1941		3.18	0.07	46.28	<.001	3.042	3.311	4.18	0.08	54.68	<.001	4.029	4.329
1940–1936		3.69	0.11	33.95	<.001	3.478	3.905	4.49	0.11	39.55	<.001	4.270	4.715
All cohorts	Linear slope (*s*)	−0.24	0.01	−27.68	<.001	−0.260	−0.225	−0.31	0.01	−23.99	<.001	−0.330	−0.280
All cohorts	Quadratic slope (*q*)	−0.01	0.00	−4.47	<.001	−0.020	−0.008	−0.01	0.00	−2.81	.005	−0.020	−0.004

Abbreviations: CI, confidence interval; LB, lower bound; *SE*, standard error; UB, upper bound.

### Cohort model

Finally, the cohort model for sexual prejudice fit these data well, except for the SRMR, *χ* ^2^ (1772) = 19,805.555, *p* < .001, CFI = 0.933, RMSEA = 0.062, SRMR = 0.106. For women, Table 5 reveals that all birth cohorts showed significant quadratic changes in sexual prejudice over time (*p*s < .005). More specifically, the grey lines in Figure 1 display the birth cohorts' trends across the 11 annual assessment occasions. A visual inspection reveals visible cohort differences (as shown by the nonoverlapping 95% error bars). In particular, the younger cohorts display lower levels of sexual prejudice than their older counterparts, but all cohorts declined in their sexual prejudice at a similar rate across 11 assessment occasions. Turning to our results for men, Table 5 reveals that 11 out of 12 birth cohorts displayed significant quadratic changes in sexual prejudice over time (*p*s < .005)—only the 1995–1991 cohort displayed stable rates over time (*p*s ≥ .097). Figure 2 displays the birth cohort trends across the 11 annual assessments for men. Like women, there are visible cohort differences (shown by the nonoverlapping 95% error bars) whereby younger cohorts exhibit a lower mean level of sexual prejudice than their older counterparts. Nevertheless, all cohorts show a decline in sexual prejudice that slows over time.

**TABLE 5:** Parameter estimates for the cohort model.

Birth cohort		Women					Men				
		Est.	*SE*	*p*‐value	95% CI		Est.	*SE*	*p*‐value	95% CI	
					LB	UB				LB	UB
1995–1991	*i*	3.47	0.48	<.001	2.537	4.410	2.92	0.75	<.001	1.451	4.394
	*s*	1.97	0.47	<.001	1.044	2.890	1.08	0.74	.147	−0.378	2.531
	*q*	0.52	0.12	<.001	0.291	0.741	0.30	0.18	.097	−0.054	0.657
1990–1986	*i*	2.56	0.24	<.001	2.090	3.033	3.53	0.36	<.001	2.819	4.242
	*s*	1.26	0.31	<.001	0.653	1.873	2.04	0.47	<.001	1.126	2.961
	*q*	0.44	0.10	<.001	0.246	0.637	0.71	0.15	<.001	0.413	0.999
1985–1981	*i*	2.10	0.11	<.001	1.889	2.307	2.80	0.17	<.001	2.464	3.126
	*s*	0.85	0.20	<.001	0.455	1.243	1.33	0.32	<.001	0.705	1.960
	*q*	0.48	0.09	<.001	0.295	0.665	0.68	0.15	<.001	0.379	0.973
1980–1976	*i*	1.77	0.04	<.001	1.703	1.840	2.13	0.05	<.001	2.029	2.230
	*s*	0.31	0.10	.001	0.123	0.501	0.21	0.14	.136	−0.067	0.496
	*q*	0.44	0.08	<.001	0.275	0.602	0.41	0.12	.001	0.161	0.648
1975–1971	*i*	1.78	0.02	<.001	1.732	1.821	2.24	0.03	<.001	2.175	2.300
	*s*	−0.22	0.03	<.001	−0.271	−0.169	−0.26	0.04	<.001	−0.335	−0.190
	*q*	0.54	0.07	<.001	0.395	0.682	0.46	0.11	<.001	0.250	0.663
1970–1966	*i*	2.09	0.02	<.001	2.039	2.134	2.68	0.03	<.001	2.614	2.743
	*s*	−0.69	0.06	<.001	−0.814	−0.566	−0.98	0.09	<.001	−1.151	−0.806
	*q*	0.47	0.07	<.001	0.343	0.605	0.64	0.09	<.001	0.459	0.818
1965–1961	*i*	2.71	0.06	<.001	2.600	2.822	3.44	0.07	<.001	3.296	3.586
	*s*	−1.27	0.12	<.001	−1.498	−1.043	−1.63	0.15	<.001	−1.928	−1.333
	*q*	0.51	0.06	<.001	0.396	0.632	0.66	0.08	<.001	0.507	0.815
1960–1956	*i*	3.58	0.12	<.001	3.341	3.825	4.43	0.15	<.001	4.130	4.734
	*s*	−1.74	0.17	<.001	−2.077	−1.411	−2.08	0.21	<.001	−2.497	−1.667
	*q*	0.49	0.06	<.001	0.376	0.603	0.59	0.07	<.001	0.447	0.730
1955–1951	*i*	4.95	0.27	<.001	4.435	5.473	6.51	0.31	<.001	5.894	7.118
	*s*	−2.63	0.27	<.001	−3.164	−2.102	−3.47	0.32	<.001	−4.094	−2.845
	*q*	0.59	0.07	<.001	0.455	0.724	0.79	0.08	<.001	0.632	0.948
1950–1946	*i*	5.94	0.61	<.001	4.740	7.132	7.77	0.69	<.001	6.416	9.119
	*s*	−2.85	0.51	<.001	−3.844	−1.853	−3.66	0.58	<.001	−4.788	−2.523
	*q*	0.52	0.11	<.001	0.315	0.725	0.69	0.12	<.001	0.455	0.925
1945–1941	*i*	5.63	1.22	<.001	3.235	8.018	7.68	1.36	<.001	5.001	10.349
	*s*	−2.03	0.84	.016	−3.688	−0.381	−2.65	0.95	.005	−4.504	−0.788
	*q*	0.31	0.15	.032	0.027	0.594	0.37	0.16	.022	0.053	0.693
1940–1936	*i*	9.50	2.78	.001	4.064	14.941	8.88	2.70	.001	3.597	14.172
	*s*	−3.77	1.65	.022	−7.000	−0.540	−3.20	1.60	.046	−6.346	−0.060
	*q*	0.52	0.24	.033	0.041	0.994	0.46	0.24	.050	0.000	0.928

Abbreviations: CI, confidence interval; *i*, intercept; LB, lower bound; *q*, quadratic slope; *s*, linear slope; *SE*, standard error; UB, upper bound.

Given that ageing, period and cohort models all fit these data well, a combination of the processes likely contributes to the development of sexual prejudice. A visual inspection of the ageing and cohort effects in Figures 1 and 2 demonstrates that, although the cohort and ageing models overlap at points across the adult lifespan, this overlap is rare—particularly among the older cohorts (1960–1936). Moreover, there is minimal overlap between the cohorts for both men and women (as shown by the nonoverlapping 95% error bars), especially among the older cohorts. Although each cohort displays different initial mean levels of sexual prejudice, all cohorts display a similar rate of change in their sexual prejudice over time. This suggests that, rather than normative development, shared social conditions are likely responsible for the declines in sexual prejudice observed among men and women of all ages over time.

## DISCUSSION

The current pre‐registered study utilized 11 annual waves of longitudinal panel data and cohort‐sequential latent growth curve modelling to examine the growth trajectories of sexual prejudice across the adult lifespan. In doing so, we are the first to illuminate whether the well‐known generational differences in sexual prejudice reflect a normative ageing process (e.g., Peterson et al., 2020; Wilson, 1996) or cohort differences that emerge due to the different cultural zeitgeist in which each generation reached maturity (Crandall et al., 2018; Sherif & Sherif, 1953). Given the importance of social norms in fostering prejudice (Aksoy et al., 2020; Matthews, 2005) and the robust support for the impressionable years' hypothesis (Alwin et al., 1991; Osborne et al., 2011; Sears, 1981), we anticipated to find cohort‐based differences in sexual prejudice (Hypothesis 1).

Consistent with the well‐established generational gap in sexual prejudice (Herek, 2009; Herek & McLemore, 2013), our results reveal that older generations have higher initial mean levels of sexual prejudice than their younger counterparts. At first blush, these results could be mistaken for normative ageing. However, the cohort *and* ageing models rarely overlap, especially among older birth cohorts. Thus, as predicted, we reveal discernible cohort differences in sexual prejudice. Specifically, we show that cohorts differ in their initial mean levels of sexual prejudice. But, contrary to the impressionable years hypothesis, cohorts display comparable rates of change in sexual prejudice over time. These data suggest a clear *period effect* with comparable and significant declines in sexual prejudice for all birth cohorts for women and 11 (out of 12) birth cohorts for men.

That the majority of cohorts are declining in their sexual prejudice, despite coming of age with vastly different group norms, affirms that people's attitudes are malleable across the adult life span (Crandall et al., 2018; Sherif & Sherif, 1953). Thus, our results support the lifelong openness model (Sears, 1983; Tyler & Schuller, 1991) and norm‐based explanations of sexual prejudice (e.g., Álvarez‐Benjumea, 2025; Crandall et al., 2018) to suggest that changes in shared social conditions provide all cohorts the opportunity to update their prejudices and conform to group norms. Therefore, elucidating the development of prejudiced attitudes requires a shift from rigid age‐based explanations towards a more flexible approach which accounts for changes in the broader socio‐political environment (also see Tyler & Schuller, 1991). Doing so is particularly important as prejudiced norms wax and wane across time and space (Arnold et al., 2026; Crandall et al., 2018; Haas & Lannutti, 2024).

### Gender differences in sexual prejudice

We also examined potential gender differences in both the mean levels of, and rates of change in, sexual prejudice. As hypothesized, men reported higher mean levels of sexual prejudice than women. These results are consistent with the assumption that men are more motivated to endorse sexual prejudice because doing so emphasizes their masculinity and subsequent place in the social hierarchy (see Kite & Whitley, 1997). Although we anticipated that men would also report slower rates of change in sexual prejudice than women (Hypothesis 2), we found that men and women display comparable rates of change in sexual prejudice across time. In this case, previous scholars have (most likely) overestimated the rigidity of masculine socialization and sexual prejudice across the adult lifespan (c.f., Poteat & Anderson, 2012). Although beyond the scope of the present study, these results imply that (most) men are aware of the social sanctions for expressing sexual prejudice in the 21st century (Álvarez‐Benjumea, 2025; Crandall et al., 2018) and, thus, adopt more progressive attitudes towards sexual minorities.

### Strengths, policy implications and future research directions

By utilizing 11 annual waves of longitudinal panel data containing responses from over 60,000 participants, the current study provides the most comprehensive picture of the development of sexual prejudice across adulthood to date. Indeed, given that it is impractical, if not impossible, to obtain data spanning an entire lifetime, a cohort‐sequential latent growth curve model is the most effective approach to separating and comparing ageing, period and cohort effects across the adult life span (Lilly et al., 2025; Zubielevitch et al., 2023). Our exceptionally large sample size and unique analytic method also allow us to accurately identify meaningful, albeit small, effects that would otherwise be unreliable in an underpowered study (Götz et al., 2022). And although they require careful interpretation, “small” effects are becoming the norm in psychology—especially when charting the lifespan development of complex attitudes (Götz et al., 2022). For example, prior research utilizing the same dataset and analytic approach reveals comparable effect sizes when assessing the developmental trajectories of right‐wing authoritarianism and social dominance orientation (Zubielevitch et al., 2023), individual‐ and group‐based relative deprivation (Lilly et al., 2025), Gender Identity Centrality (Hill Cone et al., 2025) and climate change beliefs (Milfont et al., 2021). Thus, this analytic strategy clarifies for the first time that initial mean levels—but not the trajectory—of sexual prejudices differ among cohorts and genders. Our results thus highlight the lifetime malleability of sexual prejudice and raise important implications and future research directions for the sexual prejudice literature.

Practically, (even small) declines in sexual prejudice across cohorts can translate to important political consequences (also see Götz et al., 2022). Not long ago, same‐sex marriage was a ‘hot button issue’, and politicians were reluctant to support LGBTQIA+ rights (Baunach, 2012). Numerous politicians, including former U.S. President Barack Obama, argued that their initial opposition to LGBTQIA+ rights was not reflective of their personal values, but rather, was because same‐sex marriage was a political non‐starter (McCarthy, 2015). However, the decline in sexual prejudice across cohorts observed here suggests that public support for the LGB+ community is politically advantageous in 2026. Consistent with this perspective, members of the National Party (e.g., New Zealand's centre‐right party) directly attribute their recent support for same‐sex marriage to the discernible decline in sexual prejudice among New Zealanders (Yeoman, 2019). We, therefore, caution against prematurely dismissing small declines in sexual prejudice, and reassure politicians that support for LGBTQIA+ rights is not only tolerated but expected—at least among New Zealanders.

Although our cohort‐sequential latent growth curve model captures a *mean‐level* decline in sexual prejudice across cohorts of men and women, multiple conservative governments, including the Trump administration, were recently elected despite their political campaigns being centred on censoring LGBTQIA+ activism and erasing LGBTQIA+ history (Arnold et al., 2026; Crandall et al., 2018). Likewise, despite ostensible trends towards egalitarianism, New Zealand has witnessed an unprecedented rise in anti‐LGBTQIA+ protests and hate crimes in recent years (Daalder, 2022). Therefore, (at least) a small subgroup of the population remains high or is increasing in their sexual prejudice across time. For instance, although trajectories in sexual prejudice may not vary by generational cohort or gender, they may differ as a function of other demographics (e.g., religiosity) and ideology (political conservatism). We strongly encourage future scholars to employ person‐centred analysis, such as Latent Class Growth Curve analysis, to elucidate the (potentially) heterogeneous growth trajectories in sexual prejudice, as well as the socio‐demographic factors which may influence their rates of change over time.

It is also worth noting that, because we utilize a single‐item measure of sexual prejudice, we cannot capture the different components of prejudice nor perform measurement invariance tests over time. Thus, responses to our item could reflect changes in the normative climate or reinterpretation of our item rather than enduring changes in affect, cognition, or behaviour towards the LGB+ community. For example, some cohorts may feel disgust (affective component), act violently (behavioural component) or endorse negative stereotypes (the cognitive component) towards sexual minorities but understand that it is socially inappropriate to express these prejudices. Although it may be tempting to dismiss our findings on these grounds, the different components of sexual prejudice are highly correlated. Indeed, those who self‐report acceptance of homosexuality are also likely to vote for pro‐LGB+ policies and feel warm towards their LGB+ peers (Chonody, 2013; Clarke, Sibley, et al., 2026; Herek, 2009). Additionally, our single item has high face validity and is a well‐utilized measure of sexual prejudice across cultures (Poushter & Kent, 2020). Our results thus indicate that cohorts are sensitive to changes in social norms and, at the very least, adjust their explicit attitudes accordingly. In other words, although we cannot ascertain if our results reflect a true change in people's hearts, they do indicate an important change in people's minds.

Despite drawing on an exceptionally large sample of over 60,000 people, there are three sample constraints worth considering. First, supplementary analysis reveals some evidence of selective attrition (see Table S1 in the Online Supplementary Materials). Although it is important to acknowledge selective attrition in longitudinal panel studies (Satherley et al., 2015), it is unlikely to produce the complex pattern of results observed here (see OSM for further discussion). Second, our sample of TGD participants was too small to be included in our multi‐group analyses. This sample constraint is particularly unfortunate, considering the growing calls to examine TGD people as political actors who hold unique attitudes towards LGB+ issues (Egan, 2025). For instance, given that TGD people typically report more acceptance of LGB+ people than their cisgender counterparts (Fisher et al., 2017), cohorts of TGD people likely display distinct initial mean levels and growth trajectories of sexual prejudice. Finally, we overrepresent women in the current study. Although this gender imbalance is mitigated by our multi‐group analysis which estimated the growth trajectories separately for men and women, future research would benefit from a more representative sample of genders, including an explicit exploration of TGD people's sexual prejudice.

Finally, future research should investigate the mechanism(s) responsible for the decline in sexual prejudice across cohorts. Although we attribute the declines in sexual prejudice to changes in shared social conditions and norms, we cannot directly determine what catalysed these changes. That is, our analysis reveals *when* change occurs, but not *why*. Given that policy implementation is a powerful driver of social norms (Álvarez‐Benjumea, 2025), it is possible that the recent passage of progressive policies facilitated the decline in sexual prejudice across cohorts and genders observed here. Consistent with this perspective, emerging research reveals that support for same‐sex marriage precedes declines in sexual prejudice for some groups in New Zealand (Clarke, Sibley, et al., 2026). Alternatively, pro‐LGB+ norms may be achieved through incremental steps and the cumulative efforts of activists and politicians over the past decades. Although beyond the scope of the present study, elucidating exactly *why* sexual prejudice is declining over time is essential to understanding when and how people will adopt progressive attitudes towards sexual minorities.

## CONCLUSION

The current study examined whether generational differences in sexual prejudice reflect a process of normative ageing or differences across cohorts. Specifically, we leveraged 11 annual waves of longitudinal panel data to conduct a series of cohort‐sequential latent growth curve models that test for ageing, period and cohort effects in sexual prejudice across the adult life span (i.e., 19–84 years of age) among men and women. Although our results reveal that cohorts and genders displayed different initial mean levels of sexual prejudice, almost all cohorts declined in sexual prejudice at a similar rate over time. Thus, despite the assumed rigidity of attitudes later in life (Alwin et al., 1991; Osborne et al., 2011), our results indicate that sexual prejudice remains malleable across the lifespan for both men and women. As such, we echo scholars' optimism for the future of LGBTQIA+ rights and argue that changes in shared social conditions can facilitate a decline in sexual prejudice across cohorts and genders.

## AUTHOR CONTRIBUTIONS

**EVC**: Conceptualization (lead), Formal analysis, Writing – original draft (lead), Writing – review & editing, Funding Acquisition, visualization. **CGS**: Conceptualization (supporting), Data Curation, Funding Acquisition, Writing – review and editing, Supervision. **DO**: Conceptualization, Writing – original draft (supporting), Supervision.

## CONFLICT OF INTEREST STATEMENT

The authors confirm that we have no potential financial or non‐financial conflicts of interest to declare.

## Supporting information


**Table S1.** Analyses examining differences between consistent and inconsistent responders on focal variables.

## Data Availability

The data described in our paper are derived from the New Zealand Attitudes and Values Study (NZAVS), an ongoing, nationwide longitudinal panel study. The NZAVS is approved by the University of Auckland Participants Ethics Committee (Reference Number: UAHPEC22576) and is in accordance with the established ethics standards. Our ethics approval, which dates back to 2009 (and has been updated every 3 years), specifies that we are unable to post our dataset online—doing so would violate the conditions of our ethics approval. However, full copies of the NZAVS data files are held by all members of the NZAVS management team and advisory board. A de‐identified dataset containing the variables analysed in this manuscript is available upon request from the corresponding author or any NZAVS advisory board member for replication or checking any published study using NZAVS data. The design and analysis for the current study were pre‐registered on the Open Science Framework (OSF): https://osf.io/x4mb5. All study materials are publicly available: https://osf.io/75snb/. All analysis scripts are publicly available: https://osf.io/dz92p. No artificial intelligence–assisted technologies were used in this research or the creation of this article.

## Footnotes

^1^
During the review process, another wave of data became available (Time 16), which we now incorporate into our revised manuscript.
