# Supplementary material for: Sexual prejudice declined across generational cohorts and genders: A cohort sequential latent growth curve model from 2014 to 2024
Source: Br J Soc Psychol. 2026 Apr 28;65:e70087. doi: 10.1111/bjso.70087 (PMC13123201; doi:10.1111/bjso.70087)
Supplement: Supplementary file 1 — Table S1. Analyses examining differences between consistent and inconsistent responders on focal variables. [file BJSO-65-0-s001.docx]

**Sexual Prejudice Declined Across Generational Cohorts and Genders: A Cohort Sequential Latent Growth Curve Model From 2014 to 2024**

Online Supplementary Materials

**Table S1**

*Analyses Examining Differences Between Consistent and Inconsistent Responders on Focal Variables*.

|  | Consistent Responder  (*N* = 14,768) | |  | Withdrew / Inconsistent  (*N =* 48,790) | |  | Mean Difference | | | |
| --- | --- | --- | --- | --- | --- | --- | --- | --- | --- | --- |
|  | *M* | (*SD*) |  | *M* | (*SD*) |  | *M* | *t* | *df* | *p* |
| Male | 0.37 | (0.48) |  | 0.37 | (0.48) |  | 0.00 | 0.03 | 63556 | .978 |
| NZ European | 0.96 | (0.19) |  | 0.89 | (0.31) |  | 0.07 | 25.81 | 63556 | < .001 |
| Māori | 0.09 | (0.28) |  | 0.14 | (0.34) |  | -0.05 | 16.38 | 63556 | < .001 |
| Pacific | 0.02 | (0.15) |  | 0.04 | (0.19) |  | -0.02 | 8.77 | 63556 | < .001 |
| Asian | 0.04 | (0.19) |  | 0.07 | (0.25) |  | -0.03 | 13.25 | 63556 | < .001 |
| Age | 51.02 | (12.53) |  | 45.37 | (13.16) |  | -0.02 | 46.27 | 63556 | < .001 |
| Sexual Prejudice_T6_ | 2.32 | (1.67) |  | 2.46 | (1.78) |  | -0.14 | 4.05 | 14915 | < .001 |
| Sexual Prejudice_T7_ | 2.26 | (1.68) |  | 2.40 | (1.76) |  | -0.13 | 3.82 | 13282 | < .001 |
| Sexual Prejudice_T8_ | 2.18 | (1.61) |  | 2.31 | (1.71) |  | -0.13 | 4.49 | 20447 | < .001 |
| Sexual Prejudice_T9_ | 2.16 | (1.67) |  | 2.20 | (1.69) |  | -0.04 | 1.31 | 16164 | .189 |
| Sexual Prejudice_T10_ | 2.07 | (1.61) |  | 2.22 | (1.71) |  | -0.15 | 7.90 | 43123 | < .001 |
| Sexual Prejudice_T11_ | 2.07 | (1.61) |  | 2.13 | (1.65) |  | -0.06 | 3.51 | 40397 | < .001 |
| Sexual Prejudice_T12_ | 1.87 | (1.49) |  | 1.95 | (1.56) |  | -0.08 | 4.81 | 36643 | < .001 |
| Sexual Prejudice_T13_ | 1.92 | (1.52) |  | 2.00 | (1.57) |  | -0.08 | 4.48 | 30950 | < .001 |
| Sexual Prejudice_T14_ | 1.89 | (1.49) |  | 1.99 | (1.54) |  | -0.10 | 5.38 | 29163 | < .001 |
| Sexual Prejudice_T15_ | 1.98 | (1.56) |  | 2.10 | (1.67) |  | -0.12 | 5.90 | 26609 | < .001 |
| Sexual Prejudice_T16_ | 2.01 | (1.57) |  | 2.05 | (1.57) |  | -0.05 | 2.51 | 26490 | < .012 |

**Attrition Analyses**

Given that conservatives are more likely than liberals to both distrust science (Kerr & Wilson, 2021) and harbour sexually prejudiced views (Clarke et al., 2026; Poteat & Mereish, 2012), conservative participants may have systematically withdrawn from the study. To test this possibility, we split our sample into participants who joined and consistently stayed in the study (consistent responders; *N =* 14,768) and those who responded inconsistently and/or withdrew from the study (inconsistent responders; *N =* 48,790). The *t*-tests displayed in Table 1 indicate that consistent responders were more likely to identify as New Zealand European, but less likely to identify as Māori, Pacific, and/or Asian, than inconsistent responders (*p*s < .001). Consistent responders were also significantly older (*p* < .001), but were no more likely to be male (*p* = .978), than inconsistent responders. These results largely mirror past work on the demographic correlates of panel attrition (see Satherley et al., 2015). Most importantly, inconsistent responders were higher than consistent responders on sexual prejudice at 10 of the 11 assessment waves (*p*s < .012). Thus, there is some evidence of selective attrition in our sample.

Although inconsistent responders were higher on sexual prejudice than consistent responders, this form of selective attrition is unlikely to account for the results observed in the current study for at least four reasons. First, inconsistent responders were higher on sexual prejudice across all but one of the assessments. As such, their attrition from the study would have yielded a systematic decline in sexual prejudice across assessments. Yet the ageing trend for sexual prejudice slowly increases over time. Second, the curvilinear decline in sexual prejudice begins to emerge *after* participants reach the age of 55. If this decline was due to inconsistent responders systematically leaving the study, the curvilinear decline should have emerged at younger ages, given that inconsistent responders were younger than consistent responders. Third, though significant, the mean differences in sexual prejudice across inconsistent and consistent responders are rather meagre in size. Fourth, our use of Full Information Maximum Likelihood estimates allowed us to retain the responses from inconsistent responders without biasing our estimates in ways that would exist if using alternative approaches to missing data like list-wise or case-wise deletion (see Enders & Bandalos, 2001). Thus, although there is evidence of selective attrition, it is unlikely to have produced the complex patterns observed in the current study.

**References**

Clarke, E. V., Sibley, C. G., & Osborne, D. (2026). Sexual prejudice predicts opposition to marriage equality for men and women. *Journal of Homosexuality*, *73*(2), 414–436. <https://doi.org/10.1080/00918369.2025.2475032>

Enders, C. K., & Bandalos, D. L. (2001). The relative performance of full information maximum likelihood estimation for missing data in structural equation models. *Structural Equation Modeling: A Multidisciplinary Journal*, *8*(3), 430–457. <https://doi.org/10.1207/S15328007SEM0803_5>

Kerr, J. R., & Wilson, M. S. (2021). Right-wing authoritarianism and social dominance orientation predict rejection of science and scientists. *Group Processes & Intergroup Relations*, *24*(4), 550–567. <https://doi.org/10.1177/1368430221992126>

Poteat, V. P., & Mereish, E. H. (2012). Ideology, prejudice, and attitudes toward sexual minority social policies and organizations. *Political Psychology*, *33*(2), 211–224. <https://doi.org/10.1111/j.1467-9221.2012.00871.x>

Satherley, N., Milojev, P., Greaves, L. M., Huang, Y., Osborne, D., Bulbulia, J., & Sibley, C. G. (2015). Demographic and psychological predictors of panel attrition: Evidence from the New Zealand Attitudes and Values Study. *PLoS One*, *10*(3), e0121950. <https://doi.org/10.1371/journal.pone.0121950>
